# Case Report: A new modification of Loma Linda pouch technique

**DOI:** 10.12688/f1000research.160451.1

**Published:** 2025-04-02

**Authors:** Hazem Redwan, Munir Harfouch, Abdulkarim Sheikh Ibrahim, Ali Silan, Issam Alkhouri

**Affiliations:** 1 Oral and Maxillofacial Surgery, Damascus University, Damascus, Damascus Governorate, Syria; 2 Oral and Maxillofacial Surgery, Qasyoun Private University, Ghabagheb, Daraa, Syria

**Keywords:** Bone Grafting; Collagen Membrane; Perforation; Pouch Technique; Sinus Lifting;

## Abstract

Dental implants provide a reliable treatment option for completely or partially edentulous patients. In case of a membrane perforation, the gap can be closed using a piece of resorbable collagen membrane or by suturing the Schneiderian membrane using a resorbable suture. The present study shows a new development in this technique, which involves modifying the design of the absorbable membrane and using bins to fix the membrane. This study concluded that the novel design of the collagen membrane and its fixation with the bins led to greater stability of the bone graft and led to subsequent bone gain that enables dental implantation. Still, this technique requires a histological study to determine the nature of the bone formed.

## 1. Introduction

Dental implants are the best option to compensate for partial and complete tooth loss, especially in free edentulous ridge, and they also improve the quality of life for individuals. ^1^

However, they suffer from some limitations, such as important anatomical structures, which often have a lack of bone height, such as the maxillary sinus. ^2^ However, the development of many methods, such as the external sinus lift using the lateral approach, enabled us to perform dental implants when there is no bone opposite the maxillary sinus. ^2^

However, some limitations hindered the external sinus lift procedure, such as the thickness of the membrane, which may tear and hinder the completion of the external sinus lift process. ^3^Many grafting materials have been proved to enhance the antral area, such as autografts, ^2^ hydroxyapatite, ^3^ xenografts, ^4^ and various combinations of these. ^3^

Regardless of the chosen graft material, the sinus augmentation technique requires the elevation of the Schneiderian membrane and the insertion of the graft material into the area beneath the lifted membrane. ^5^

The most frequent sequela during sinus graft surgery is the tearing of the Schneiderian membrane depending on the thickness of the memebrane. ^6^

In case of a membrane perforation, the gap can be closed using a piece of resorbable collagen membrane or by suturing the Schneiderian membrane using a resorbable suture. ^7^

The Loma Linda Pouch technique was proposed by Proussaefs et al., ^8^ to create an artificial pouch from collagen membrane and the bony walls of the maxillary sinus and fill it with bone graft to solve the problem of Schneiderian membrane perforation.

Its modification was made by Das et al., ^9^ and this modification requires the membrane to be fixed at the palatal wall of the maxillary sinus and at the lateral window using sutures.

The present study shows a new development in this technique by modifying the design of the absorbable membrane and using bins to fix the membrane.

## 2. Case presentation

A non-smoker, medically free patient, aged 50 years, with need of dental implantation in the upper left area.

The patient participating in this study was informed of the details of the surgical procedure, the risks associated with it, and the alternative treatments. All his inquiries were answered and he signed the informed consent through which he agreed to enter the study and to the use of images of the surgical procedure in scientific articles.

**Figure 1.**
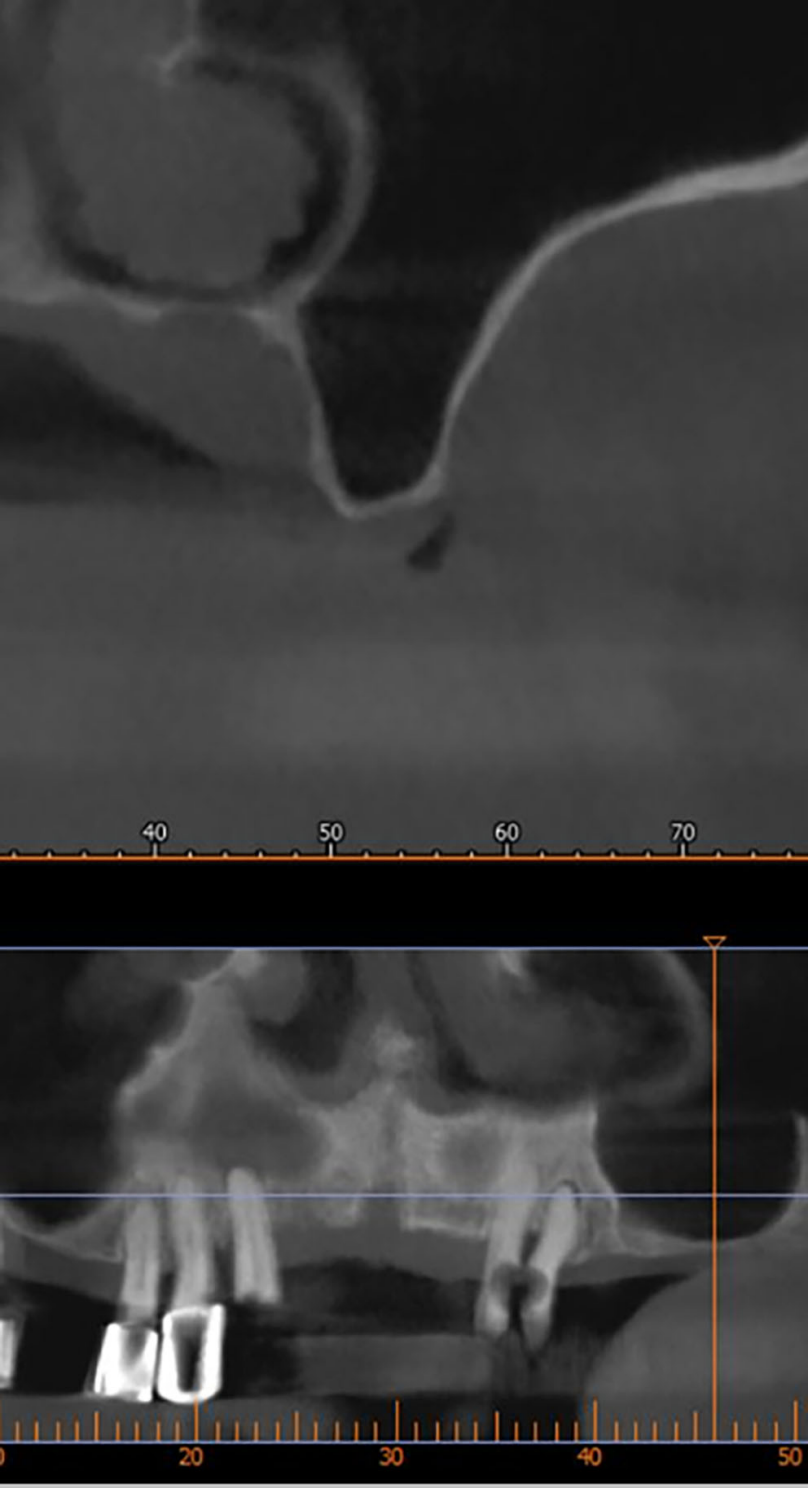
CBCT shows the thickness of the maxillary sinus floor.

After implant planning using the cone-beam computed tomography CBCT, it was found that the alveolar bone height corresponding to the maxillary sinus floor was (0.5-1 mm) ( Figure 1), which indicates an external sinus lift.

**Figure 2.**
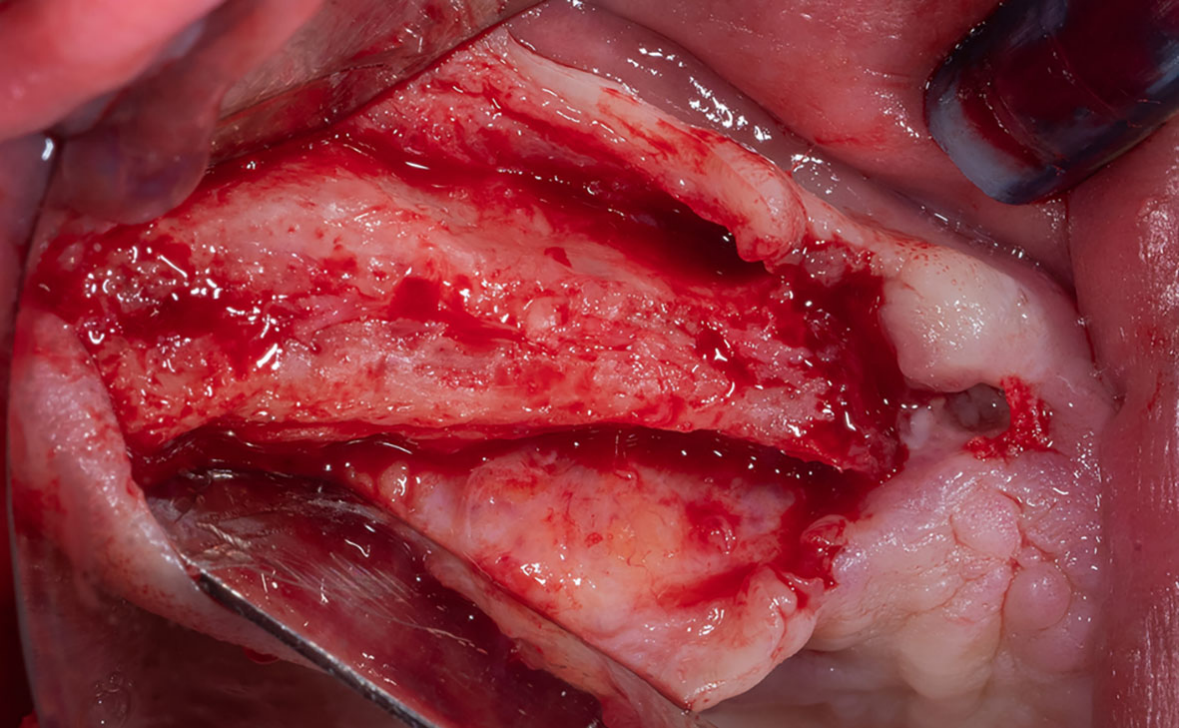
Full-thickness gingival flap lifting.

**Figure 3.**
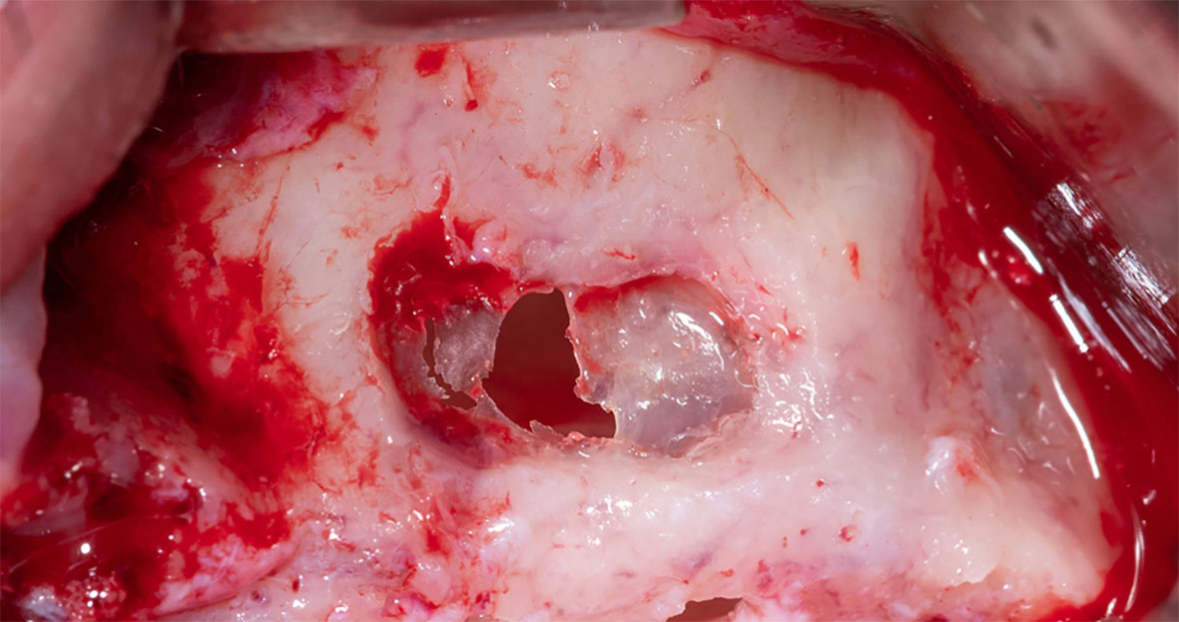
The lateral window opening using Piezo electric device.

Local anesthesia was performed by infiltration in the surgical area using 4% articaine with 1:80,000 adrenaline, a full-thickness gingival flap was lifted ( Figure 2), and a lateral window was opened in the maxillary sinus wall using Piezo electric device (PIEZOSURGERY®, Mectron, Italy) ( Figure 3).

**Figure 4.**
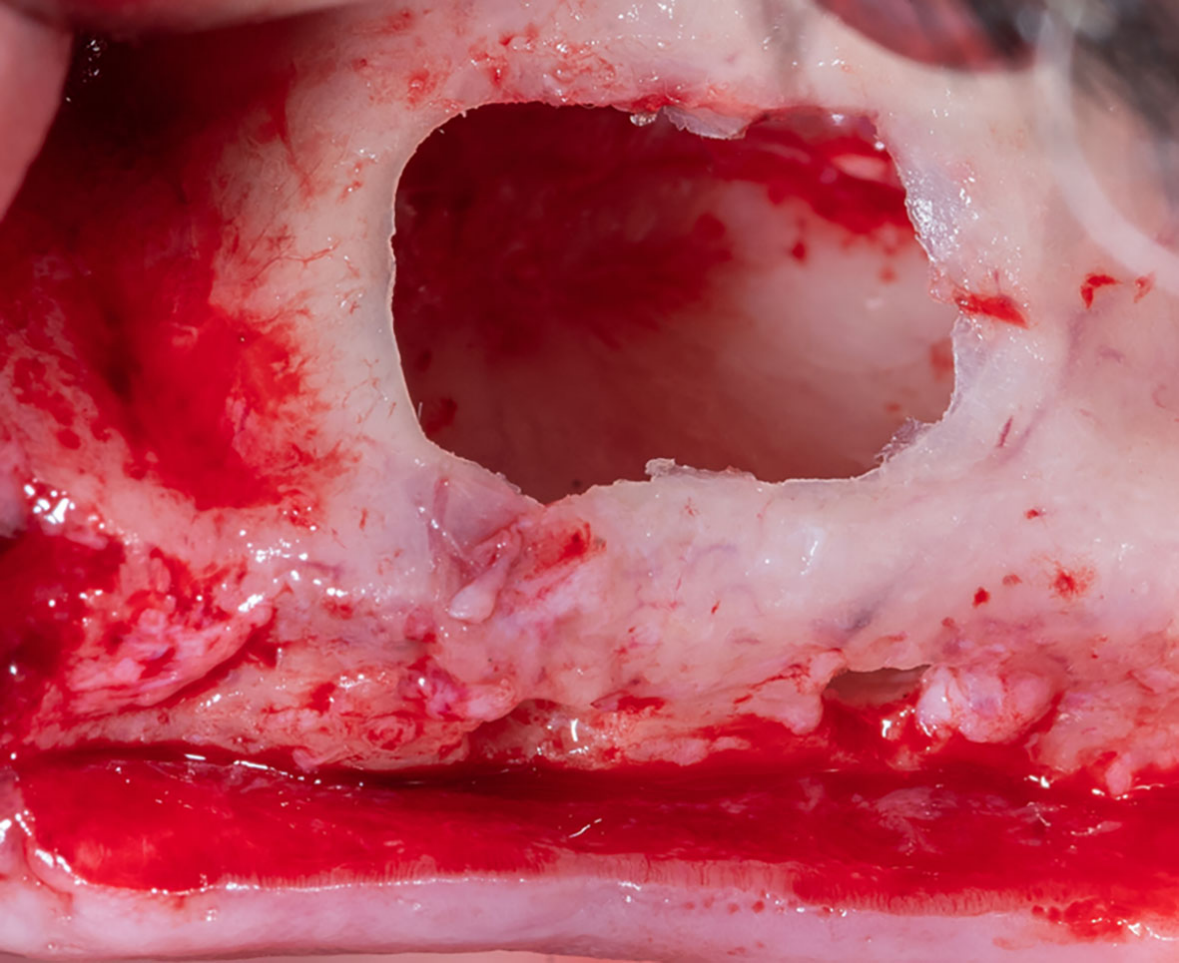
The final window after lifting schneiderian membrane.

**Figure 5.**
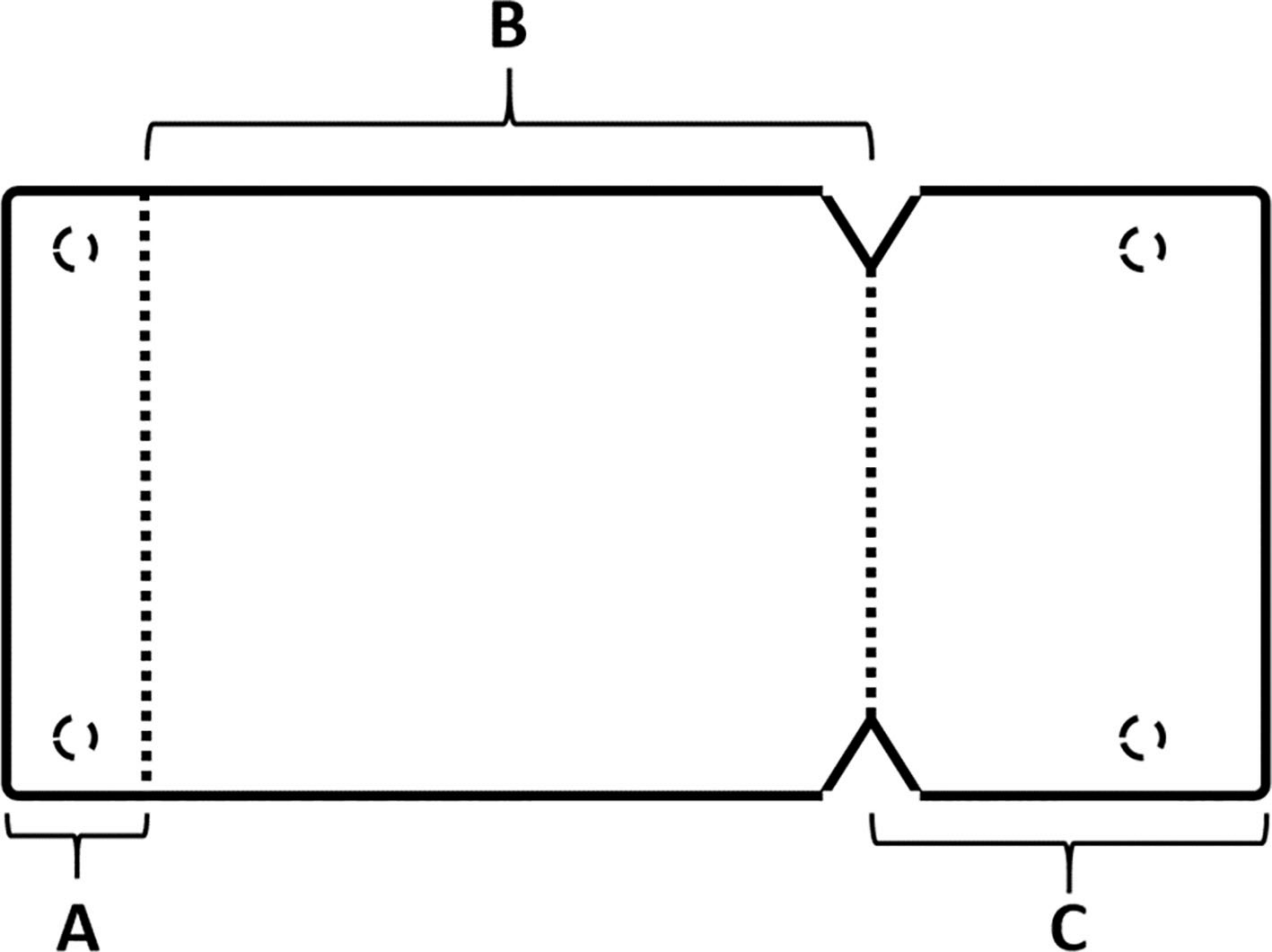
The novel design of the collagen membrane.

The sinus membrane was thin; therefore, it was torn when trying to open the lateral window and lift it, which prompted us to apply the pouch technique, which includes the following:

The membrane lifted from the inner sinus walls. Once the final intervention window is prepared ( Figure 4), we prepare the absorbable collagen membrane (Jason® membrane, Botiss, Germany) using the modified design performed by the researcher as it was used in this study for the first time ( Figure 5), where the membrane is measured and divided imaginarily into three parts as follows:

A: 4 mm wide, faces the palatal wall, and is fixed with it using pins.
B: the same width as the maxillary sinus’s buccal palatal width and forms the roof of the artificially formed pouch.
C: 2 mm larger than the lateral window.

**Figure 6.**
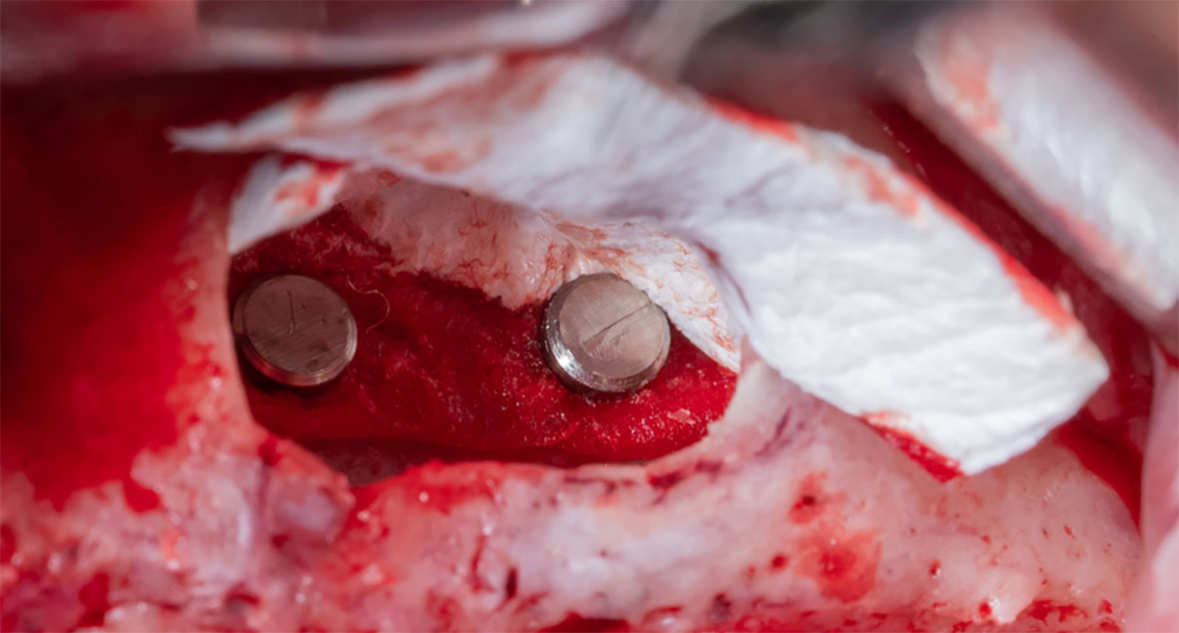
Fixing the collagen membrane to the palatal wall using pins.

Part A is determined and fixed to the palatal wall of the maxillary sinus using 2 pins (titan pin®, Botiss, Germany) and with a height equal to the future implants’ length + 2 mm ( Figure 6).

**Figure 7.**
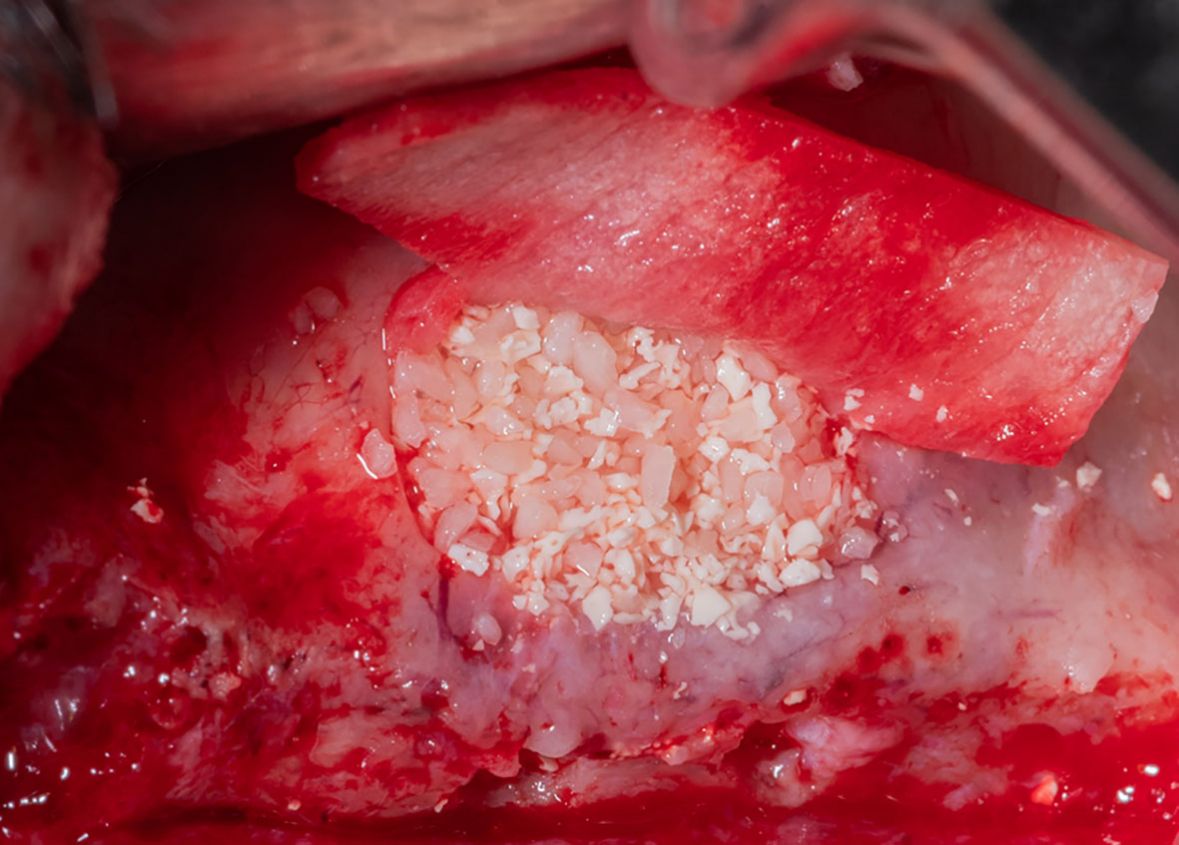
Filling the formed pouch with the bone graft mixture.

After fixing the membrane to the palatal wall, the formed pouch is filled with a mixture of allograft (MYBONE, MSBIO, Korea) and xenograft (cerabone, Botiss, Germany) (30:70) ^10^ until it is filled ( Figure 7). Part B forms the roof of the formed pouch, compensating for the torn sinus membrane.

**Figure 8.**
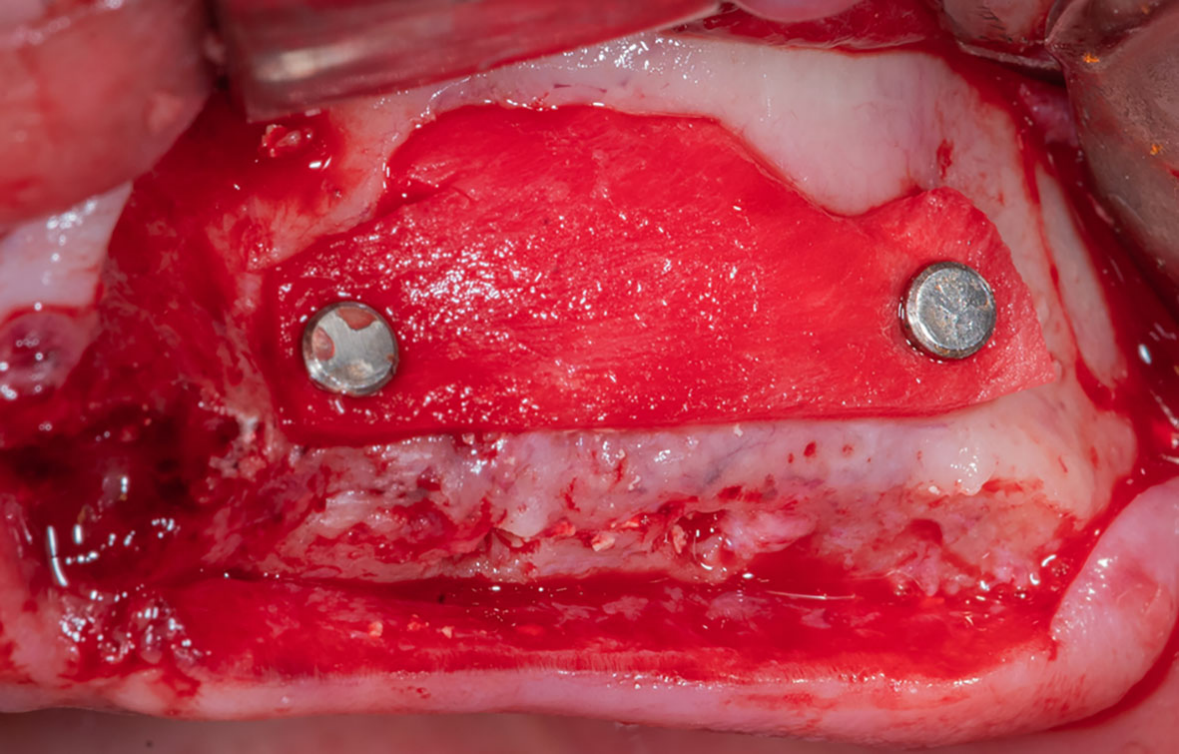
Fixing the collagen membrane using pins to close the lateral window.

After that, the lateral window is closed with part C of the collagen membrane and fixed using pins medial and lateral to the window ( Figure 8), each pin is 2 mm away from the window border.

Mechanical fixing of the membrane using pins and filling the formed pouch contributes to bone graft stability in the formed sinus and prevents the displacement of both the membrane and the bone graft mixture.

**Figure 9.**
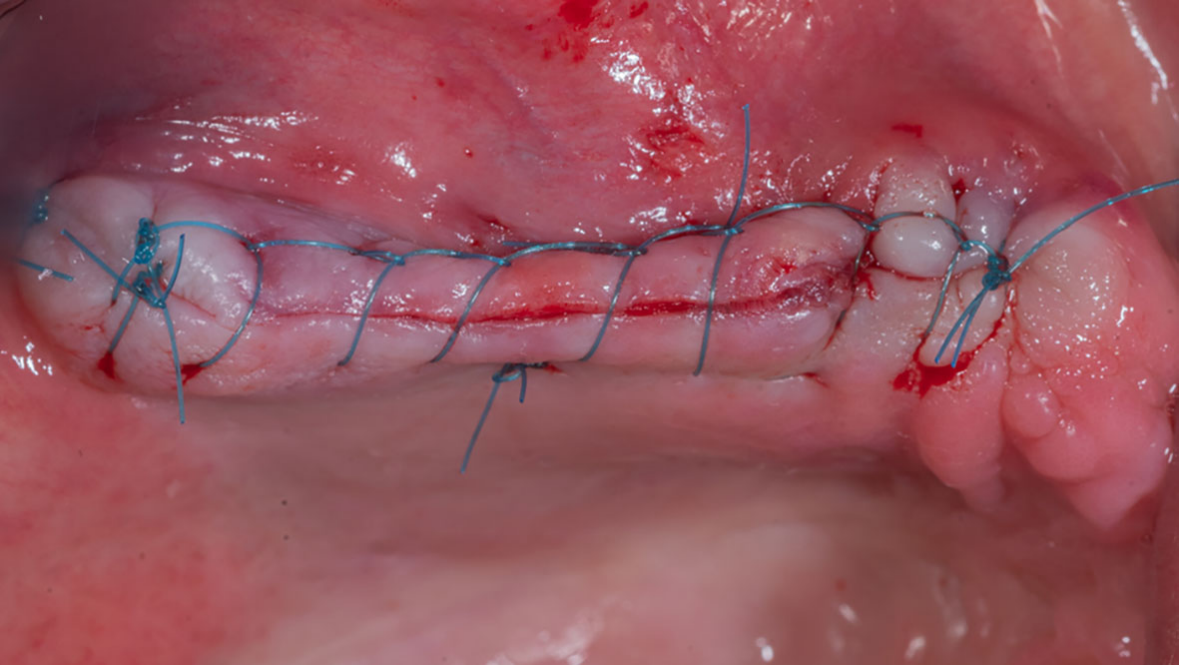
The suture of the gingival flap.

The gingival flap is then repositioned and sutured ( Figure 9). The patient was given postoperative instructions including not to rinse for 24 hours and to follow a soft diet during the first week after surgery. The patient was also given oral hygiene instructions.

An antibiotic (Amoxicillin 875 mg and Clavulanic acid 125 mg) was prescribed every 12 hours for 7 days and Diclofenac potassium 50 mg when needed, not to exceed 150 mg daily, in addition to chlorhexidine 0.12% mouthwash twice daily, 60 seconds for 14 days starting a day after surgery.

The surgical sutures were removed 14 days after surgery, and no sequelae occurred during the follow-up period that extended to nine months.

**Figure 10.**
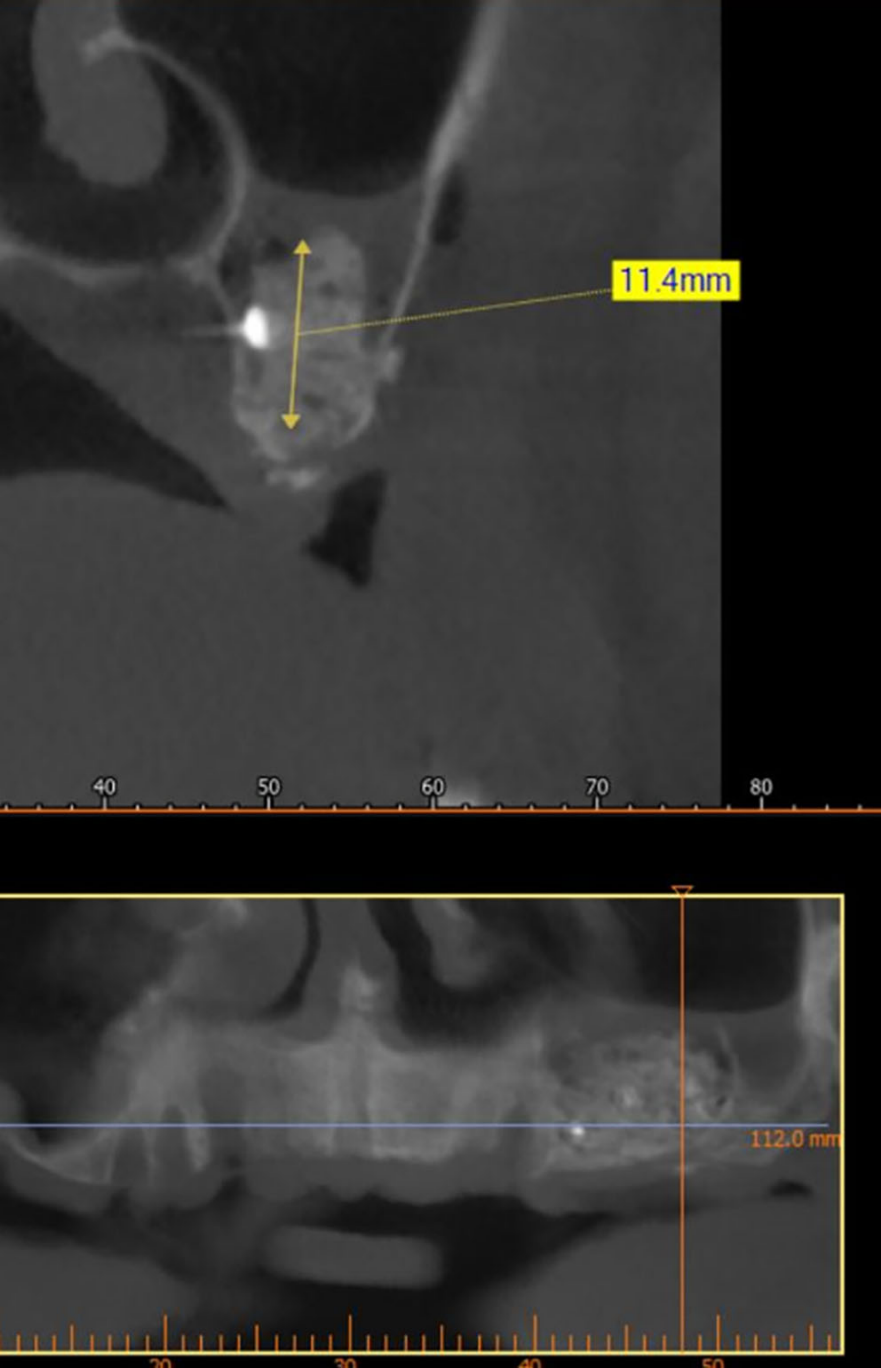
CBCT after grafting immediately.

**Figure 11.**
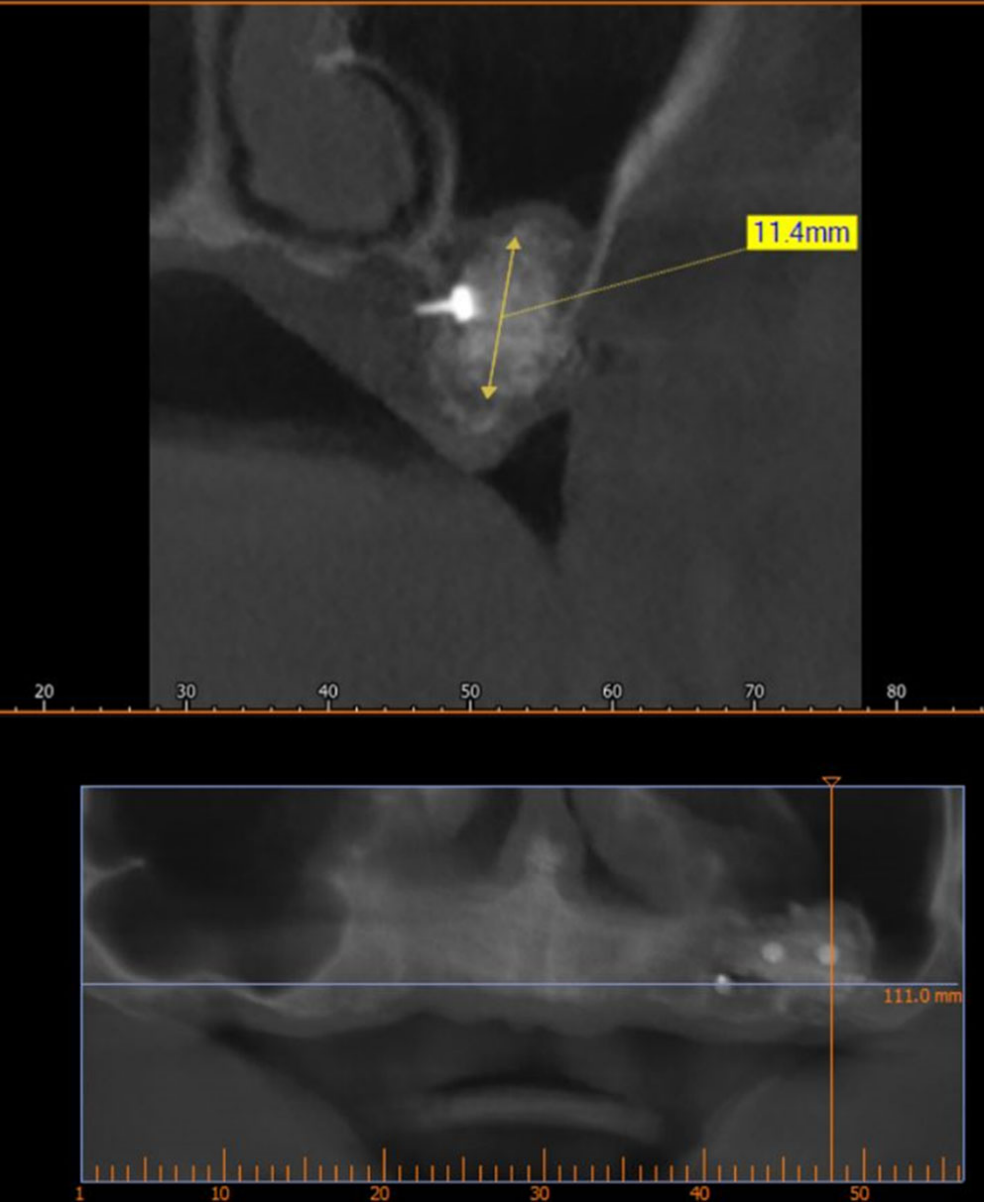
CBCT 9 months after grafting.

A CBCT scan was performed immediately after grafting and showed a direct bone gain of (11.4 mm) ( Figure 10) and 9 months after bone grafting where subsequent bone gain (11.4 mm) was found ( Figure 11).

## 3. Discussion

Schneiderian membrane’s perforation is the most frequent sequela seen during sinus grafting. ^11^ Jensen et al., ^12^ report an occurrence of Schneiderian membrane perforation in 35% of cases, while others ^13^ report a 7% to 56% frequency when sinus grafting.

Several studies have advised the use of a resorbable collagen membrane to repair the perforated sinus membrane. ^14^

The aforementioned pouch technique may provide high protection and isolation of the graft material when conducting sinus grafting procedures. Furthermore, the creation of a pouch in the aforementioned technique presents the opportunity to cover the lateral access window with the collagen membrane employed to close the perforated area. This may cause increased isolation of the graft material. ^9^

Enhanced bone formation was observed in the grafted maxillary sinus when a non-resorbable barrier was applied to protect the graft material. However, other authors have suggested the resorbable collagen membrane use. ^15^

The Loma Linda pouch technique may provide enhanced isolation and protection of the graft material in sinus grafting procedures, particularly in cases where a perforation of the sinus membrane has occurred. ^8^

In the original proposal for the Loma Linda Pouch technique, ^8^ the collagen membrane is left unfixed and is stabilized by filling the pouch with bone graft, while in, ^9^ it is stabilized using sutures. This differs from the current study, which relied on a membrane design such that it is stabilized with bins while increasing the bone contact between the graft material and the bone walls.

However, a histological study is required to include histologic specimens that demonstrate bone formation within the sinus area.

## 4. Conclusion

Among the limitations of the current study, the novel design of the collagen membrane and its fixation with the bins led to greater stability of the bone graft and led to subsequent bone gain that enables dental implantation, but this technique requires a histological study to determine the nature of the bone formed.

## Ethics approval and consent to participate

This study is approved by the ethical committee of Damascus University with number (DN-DN-020624-235). The study was registered on 5/16/2021 at Damascus University and the study start date was 9/21/2021. The patient participating in this study was informed of the details of the surgical procedure, the risks associated with it, and the alternative treatments. All his inquiries were answered and he signed the written informed consent through which he agreed to enter the study and to the use of images of the surgical procedure in scientific articles.

## Consent to participate

The patient participating in this study was informed of the details of the surgical procedure, the risks associated with it, and the alternative treatments. All his inquiries were answered and he Written informed consent through which he agreed to enter the study and to the use of images of the surgical procedure in scientific articles.

## Data Availability

No data are associated with this article
