# Supplementary material for: Case Report: A new modification of Loma Linda pouch technique
Source: F1000Res. 2025 Aug 22;14:383. Originally published 2025 Apr 2. [Version 3] doi: 10.12688/f1000research.160451.3 (PMC12082067; doi:10.12688/f1000research.160451.3)
Supplement: Peer review history

# Reviewer report: Reviewer response for version 3

**DOI:** 10.5256/f1000research.186718.r407885

**Published:** 2025-08-30

**Authors:** Mhd Bahaa Alhaffar

**Affiliations:** 1 Karolinska institute, Stockholm, Sweden

the revised version has addressed all the comments and suggestion, i recommend to index the manuscript.

Are enough details provided of any physical examination and diagnostic tests, treatment given and outcomes?

Yes

Is the case presented with sufficient detail to be useful for other practitioners?

Yes

Is sufficient discussion included of the importance of the findings and their relevance to future understanding of disease processes, diagnosis or treatment?

Yes

Is the background of the case’s history and progression described in sufficient detail?

Yes

Reviewer Expertise:

dental research, dental surgery, oral health, periodontics, dental implants

I confirm that I have read this submission and believe that I have an appropriate level of expertise to confirm that it is of an acceptable scientific standard.

# Reviewer report: Reviewer response for version 2

**DOI:** 10.5256/f1000research.180711.r381143

**Published:** 2025-05-12

**Authors:** Mhd Bahaa Alhaffar

**Affiliations:** 1 Karolinska institute, Stockholm, Sweden

This case report presents a novel modification of the Loma Linda pouch technique to manage Schneiderian membrane perforation encountered during maxillary sinus augmentation. The authors propose a redesigned collagen membrane divided into three anatomical sections, fixed with titanium pins to the palatal and lateral sinus walls, to provide improved graft stability and facilitate bone regeneration. The clinical outcome was assessed via CBCT, demonstrating an 11.4 mm bone gain after nine months, although implant placement has not yet occurred. No histological examination was performed. 

 review report to be sent to the authos: 

 Dear Author,

 I had the pleasure of reading and reviewing your article. The modified Loma Linda technique you present is scientifically reasonable, and I found the clinical documentation valid and appropriate for the stated aim of the article. However, some modifications are necessary before the article can be considered for publication.

 Please justify the scientific rationale behind the specific membrane design and the chosen dimensions of its three segments (A, B, and C). The method's reproducibility across different clinical settings depends on the clarity and reasoning of these specifications. Additionally, the choice of grafting material—specifically the 70:30 xenograft-to-allograft ratio—requires more explanation. Is there evidence or experience supporting this mix in cases of sinus membrane perforation?

 This is a single case report, yet the conclusions drawn are rather strong. Without histological evaluation and without the actual placement and functional integration of implants, it is premature to claim that the technique is definitively successful. I suggest softening the conclusion and clearly stating that this case demonstrates *potential*, not confirmed clinical success. Please also revise the sentence:

 “this technique requires a histological study”

 to the more precise:

 “the nature of the regenerated tissue remains unverified in the absence of histological evidence.”

 The conclusion should be more modest and highlight the need for further studies, including histological analysis and clinical trials with larger sample sizes and follow-up post-implant placement. This will strengthen the evidence and increase the technique’s clinical validity.

 Overall, I find the article interesting and worth publishing, especially as a technical innovation. However, it would benefit from clearer justification of methods, more cautious interpretation of results, and an emphasis on the need for further research. With these small but important adjustments, the article will contribute meaningfully to the literature on sinus augmentation techniques.

 Sincerely,

 Bahaa Alhaffar

Are enough details provided of any physical examination and diagnostic tests, treatment given and outcomes?

Yes

Is the case presented with sufficient detail to be useful for other practitioners?

Yes

Is sufficient discussion included of the importance of the findings and their relevance to future understanding of disease processes, diagnosis or treatment?

Yes

Is the background of the case’s history and progression described in sufficient detail?

Yes

Reviewer Expertise:

dental research, dental surgery, oral health, periodontics, dental implants

I confirm that I have read this submission and believe that I have an appropriate level of expertise to confirm that it is of an acceptable scientific standard, however I have significant reservations, as outlined above.

# Reviewer report: Reviewer response for version 2

**DOI:** 10.5256/f1000research.180711.r381141

**Published:** 2025-05-12

**Authors:** Heba Abo-Elfetouh Elsheikh

**Affiliations:** 1 Mansoura University, Mansoura, Dakahlia Governorate, Egypt

The topic is interesting and important. However, there are some areas that need more explanation and strengthening

1. There are several instances of language inconsistencies and grammatical errors, which can affect readability. All the surgical procedures should be in the past. Improve **language and structure** through professional editing for clarity and consistency.
2. "Radiographic evaluation at the 9-month follow-up demonstrated the preservation of the grafted bone volume, with no detectable signs of bone resorption, indicating stable integration of the graft material within the augmented sinus cavity." Would you like to add potential reasons or mechanisms explaining why resorption may have been prevented in this case?
3. Nasal decongestant is beneficial in reducing mucosal edema and promoting sinus drainage—thereby potentially lowering the risk of postoperative sinusitis—there is a theoretical concern that excessive or prolonged use may transiently reduce perfusion to the surrounding tissues. However, when used appropriately in the short term, topical nasal decongestants are generally not associated with significant compromise of mucosal or graft vascularization, and are widely prescribed following sinus surgeries to support healing by maintaining sinus patency and minimizing postoperative complications." Would you like to include citations or references to support your explanation of not using nasal decongestant?
4. Can you add a live photo of the novel design of the collagen membrane?
5. Although the cross-sectional CBCT images demonstrate that the palatal pins perforated the palatal wall of the maxillary sinus, clinical examination revealed that the pins were not palpable intraorally. Would you like to include this in the discussion section of the manuscript as a clarification?
6. Will all the pins be removed during implant placement or the buccal pins only?
7. In the discussion section” while in, ^9^ it is stabilized using sutures.” Add the name of the technique. “Modified technique by Das et al.”
8. Expand the **discussion** to critically compare techniques and outcomes with those in literature.

Are enough details provided of any physical examination and diagnostic tests, treatment given and outcomes?

Partly

Is the case presented with sufficient detail to be useful for other practitioners?

Yes

Is sufficient discussion included of the importance of the findings and their relevance to future understanding of disease processes, diagnosis or treatment?

Partly

Is the background of the case’s history and progression described in sufficient detail?

Yes

Reviewer Expertise:

Oral and Maxillofacial Surgery including Oral Implantology

I confirm that I have read this submission and believe that I have an appropriate level of expertise to confirm that it is of an acceptable scientific standard, however I have significant reservations, as outlined above.

# Reviewer report: Reviewer response for version 2

**DOI:** 10.5256/f1000research.180711.r380581

**Published:** 2025-05-05

**Authors:** Lydia N. Melek

**Affiliations:** 1 Alexandria University, Alexandria, Egypt

Article can be accepted 

 Thank you

Are enough details provided of any physical examination and diagnostic tests, treatment given and outcomes?

Partly

Is the case presented with sufficient detail to be useful for other practitioners?

Yes

Is sufficient discussion included of the importance of the findings and their relevance to future understanding of disease processes, diagnosis or treatment?

Yes

Is the background of the case’s history and progression described in sufficient detail?

Yes

Reviewer Expertise:

Oral and Maxillofacial Surgery including dental impalntology

I confirm that I have read this submission and believe that I have an appropriate level of expertise to confirm that it is of an acceptable scientific standard.

# Reviewer report: Reviewer response for version 1

**DOI:** 10.5256/f1000research.176355.r375680

**Published:** 2025-04-11

**Authors:** Lydia N. Melek

**Affiliations:** 1 Alexandria University, Alexandria, Egypt

This case report provides a modification for the Loma Linda approach used during direct sinus lifting, in case of Schneiderian membrane perforation. These are some comments:

 - The writing language of the manuscript needs to be generally improved. 

 - The word "bins" needs to be replaced with "pins".

 - Use the word "mucoperiosteal flap" instead of "gingival flap".

 - Was the patient instructed to use a nasal decongestant as is usually done after sinus procedures?

 - When are implants expected to be placed? Especially with using a high percentage of xenograft that takes time in resorption?

 - Regarding the Pins placed palatally, are they palpable from the palatal side? Did they cause any irritation?

Are enough details provided of any physical examination and diagnostic tests, treatment given and outcomes?

Partly

Is the case presented with sufficient detail to be useful for other practitioners?

Yes

Is sufficient discussion included of the importance of the findings and their relevance to future understanding of disease processes, diagnosis or treatment?

Yes

Is the background of the case’s history and progression described in sufficient detail?

Yes

Reviewer Expertise:

Oral and Maxillofacial Surgery including dental impalntology

I confirm that I have read this submission and believe that I have an appropriate level of expertise to confirm that it is of an acceptable scientific standard, however I have significant reservations, as outlined above.

# Response

**Published:** 2025-04-15

**Authors:** Hazem Redwan

**Affiliations:** Oral & Maxillofacial Surgery, Damascus University, Damascus, Damascus Governorate, Syria

Dear Reviewer,

 Thanks for the precious comments that enriched our article.

 This is a Point-to-point replay,

**- The writing language of the manuscript needs to be generally improved.**

 Done.

**- The word "bins" needs to be replaced with "pins".**

 Done.

**- Use the word "mucoperiosteal flap" instead of "gingival flap".**

 Done.

**- Was the patient instructed to use a nasal decongestant as is usually done after sinus procedures?**

 No, it has not been prescribed as it will reduce blood supply in the first few days after surgery.

**- When are implants expected to be placed? Especially with using a high percentage of xenograft that takes time in resorption?**

 After 12 months.

**- Regarding the Pins placed palatally, are they palpable from the palatal side? Did they cause any irritation?**

 The pins did not palpable of the palatal and did not cause any irritation or discomfort to the patient.

 Kind regards,

 Dr. Hazem.
